# hUMSCs-exo@Cyasterone protects the cell model of steroid-induced femur head necrosis by regulating N-glycosylation modification of CTSD-N258A

**DOI:** 10.1371/journal.pone.0337562

**Published:** 2026-04-03

**Authors:** Youqiang Sun, Mengmeng Liang, Yuemeng Xing, Yifan Duan, Baogui Deng, Xiaobing Xiang

**Affiliations:** 1 Department of Sports Medicine, The First Affiliated Hospital of Guangzhou University of Chinese Medicine, Guangzhou, Guangdong, China; 2 Lingnan Medical Research Center of Guangzhou University of Chinese Medicine, Guangzhou, Guangdong, China; 3 Department of Obstetrics, Guangdong Women and Children Hospital, Guangzhou, Guangdong, China; 4 The First Clinical College of Guangzhou University of Chinese Medicine, Guangzhou, Guangdong, China; Università degli Studi della Campania, ITALY

## Abstract

**Objective:**

It has been demonstrated that both hUCMSC-exo and Cyasterone exhibit protective effects against steroid-induced osteonecrosis of the femoral head (SIONFH). Additionally, studies have shown that CTSD N-glycosylation influences BMSC apoptosis. Based on these findings, we aim to investigate the mechanism of hUCMSCs-exo@Cyasterone in the Dex-induced BMSCs model of SIONFH, focusing on its regulatory role in CTSD N-glycosylation during apoptosis.

**Methods:**

The SIONFH cell model was induced by dexamethasone (Dex) at a concentration of 10^-6^mol/L. Experiments with hUMSCs-exo@Cyasterone and CTSD mutants were performed in the BMSC model to analyze proliferation, apoptosis, lysosomal pH, lysosomal membrane permeability, and lysosomal colocalization. Additionally, the expression of apoptosis-related proteins in BMSCs and CTSD in lysosomes and the cytoplasm were examined.

**Results:**

MTT, AO staining, EDU staining, flow cytometry, and confocal microscopy revealed that hUMSCs-exo@Cyasterone attenuated the proliferation of Dex-induced BMSCs and reduced the lysosomal membrane permeability. It also decreased the expression level of apoptosis-related proteins including BID, Caspase-3, and Caspase-1, as well as the levels of CTSD in lysosomal and cytoplasm. CTSD-N258A inhibited BMSC apoptosis, enhanced the protective effect of hUMSCs-exo@Cyasterone, and promoted the lysosomal localization of CTSD and the lysosomal membrane permeability. Moreover, CTSD-N258A helped suppress the expression of apoptosis-related proteins and reduced CTSD expression in cytoplasm and lysosomes.

**Conclusion:**

hUMSCs-exo@Cyasterone mitigates apoptosis in Dex-induced BMSCs, a cell model of steroid-induced femoral head necrosis, by modulating the N-glycosylation modification of CTSD-N258A.

## Introduction

Currently, glucocorticoids stand as the foremost etiological factor in the development of osteonecrosis of the femoral head (ONFH) [1,2]. Steroid-induced necrosis of the femoral head (SIONFH) profoundly compromises patients’ quality of life and imposes substantial economic burdens [3]. Regrettably, SIONFH still lacks effective clinical therapeutic drugs, so it is necessary to find new clinical drugs for SIONFH disease.

The apoptosis hypothesis plays a pivotal role in understanding SIONFH pathogenesis [4–6]. The core of the hypothesis is that apoptosis in osteoblasts (OB), osteocytes, or bone marrow-derived stroma cells (BMSC) may eventually develop into SIONFH [2,7,8].

Cyasterone, a principal compound extracted from the root of Achyranthes bidentata, has exhibited promising efficacy in the management of osteoporosis and osteoporotic fractures [9,10]. Literature reviews have demonstrated that SIONFH apoptosis models can be established by intervening with BMSCs using dexamethasone (Dex) at a concentration of 1 × 10^-6^mol/L [8, 11–13]. Previously, our research group had done natural compounds on cell and animal experiments on SIONFH [14]. In our cellular experiments, Cyasterone was observed to mitigate Dex-induced apoptosis in BMSCs. Furthermore, in animal trials, we elucidated a methodology for generating SIONFH rat models and evaluated the protective effects of Cyasterone against SIONFH [15].

Previous studies on BMSC apoptosis have indicated that cathepsin D (CTSD) may modulate apoptosis and proliferation under diverse physiological and pathological conditions [16]. Nevertheless, its regulatory role in steroid-induced BMSC apoptosis remains unclear.

Studies have suggested that glycosylation at the 233rd asparagine (N233) residue influences pro-CTSD secretion, with ecdysone promoting CTSD expression, inducing CTSD maturation via autophagy, and stimulating apoptotic protease Caspase-3 activation, thereby fostering larval midgut cell apoptosis. It is predicted that there are N-glycosylation modification sites in both human and rat CTSD proteins. The N-glycosylation determines the secretion of CTSD zymogen, induces CTSD maturation, and finally promotes apoptosis [16–18]. What’s more, it has also been found that human umbilical mesenchymal stem cell exosomes (hUMSCs-exo) can prevent SIONFH by inhibiting osteocyte apoptosis [19–21].

Above all, we found that both the hUCMSC-exo and Cyasterone have protective effects on SIONFH disease. On the other hand, the previous study showed that CTSD N-glycosylation influences BMSC apoptosis. Based on this, we propose the scientific hypothesis for this study: “hUCMSCs-exo@Cyasterone may modulate Dex-induced BMSC apoptosis, the SIONFH cell model, by regulating CTSD N-glycosylation modification levels, ultimately ameliorating the SIONFH cellular model.”

## Materials and methods

### Exosome isolation and characterization

Human umbilical mesenchymal stem cells (hUMSCs) were purchased from Procell Life Science & Technology Co.,Ltd. (Wuhan, China) and cultured in the complete culture medium (CM-CL11, Procell) in an incubator (with 5% CO2 at 37°C). hUMSC-derived exosomes were isolated from 48 h serum-free conditioned medium using the Total Exosomes Isolation Kit (4478359, Invitrogen, USA). Briefly, the conditioned medium was centrifuged at 2,000 rpm for 20 min to remove cells and debris, followed by filtration through a 0.22 μm membrane. Exosome precipitation reagent was added to the clarified supernatant at a 1:4 ratio, vortexed thoroughly, and incubated at 4°C overnight. Exosomes were pelleted by centrifugation at 10,000g for 1 h at 4°C. Isolated exosomes were characterized by transmission electron microscopy (TEM; HT7700, HITACHI, Japan) for morphology and by dynamic light scattering (DLS; Mastersizer 3000, Malvern, UK) for particle size distribution analysis. The biomarkers of exosomes were also analyzed by using Western Blot assay.

### Preparation of hUMSCs-exo@Cyasterone

Cyasterone (HY-N0211, MCE, USA) was dissolved in DMSO to a stock concentration of 75 µg/mL. hUMSC-derived exosomes (500 µg/mL, 100 µL) were mixed with an equal volume of the Cyasterone stock solution and subjected to pulsed sonication (bath sonicator, 40% amplitude, 120 W) on ice. The sonicated mixture was subsequently incubated at 37°C for 1 h in a water bath to restore exosomal membrane integrity. Unentrapped Cyasterone was removed by ultrafiltration (100 kDa MWCO filter) via centrifugation at 4,500 × g for 15 min. The retained Cyasterone-loaded exosomes were collected and characterized by TEM for morphological assessment, while the encapsulated Cyasterone content was quantified using high-performance liquid chromatography (HPLC; 1260 Infinity III system, Agilent, USA).

### Primary culture of BMSCs

The Sprague Dawley (SD) rat was provided by the Laboratory Animal Center of Guangzhou University of Chinese Medicine. All methods were performed in accordance with the ARRIVE guidelines and all methods were carried out in accordance with relevant guidelines and regulations. The rat was euthanized with an overdose (250 mg/kg) of pentobarbital administered intraperitoneally. BMSCs were isolated and cultured according to the previously described methods [22]. The bone marrow was flushed out from the tibia and femur of SD rats and seeded into 75 cm^2^ culture flasks with α-MEM, which contained 10% FBS and 1% penicillin-streptomycin under conditions of 5% CO_2_ and 37˚C. Every 2 ~ 3 days, the medium was changed to eliminate non-adherent cells. When the adherent cells became confluent, they were removed with 0.25% Trypsin-EDTA and passaged at a ratio of 1:2. They were characterized by flow cytometric phenotype identification using the mesenchymal stem cell criteria (Positive for CD29, and negative for CD45).

### Cell treatment

For drug treatment, P3 cells of BMSCs were trypsinized upon reaching 80% confluence, resuspended at 1 × 10⁵ cells/mL, seeded into plates, and cultured overnight (37°C, 5% CO₂, humidified atmosphere). The medium was then replaced with complete growth medium containing 1 mM dexamethasone (Dex; CT316, Beyotime, China) for 24 hours to induce differentiation. Following induction, cells were treated for 48 hours (37°C, 5% CO₂) with complete growth medium supplemented with one of the following: 2 µg/mL of Cyasterone, huMSCs-exo, huMSCs-exo@Cyasterone, or Tunicamycin (HY-A0098, MCE). For cell transfection, expression vectors encoding rat CTSD wild-type (WT) or its N143A and N258A mutants were obtained from SYNBIO TECHNOLOGIES (Suzhou, China). BMSCs were seeded the day prior to transfection to achieve 80% confluence. Cells were transfected with 1 µg of plasmid DNA (WT or mutant CTSD) using Lipofectamine™ 3000 (L3000015, Invitrogen) according to the manufacturer’s protocol. After 4–6 hours incubation with the transfection mixture, the medium was replaced with fresh complete growth medium containing or no 2 µg/mL of huMSCs-exo@Cyasterone. Transfected cells were harvested for analysis 48 hours post-transfection.

### MTT Assay

The MTT assay was employed to evaluate cell viability following the manufacturer’s protocol. Following indicated treatment, culture medium was aspirated from each well. Cells were incubated with 100 µl of MTT solution (5 mg/ml, CT316, Beyotime) for 4 hours at 37°C. The supernatant was then carefully removed, and 200 µl of DMSO was added per well to solubilize the formazan crystals. After 10 minutes of orbital shaking, absorbance was measured at 490 nm using a microplate reader (SYNERGY, BioTek, USA).

### EDU staining

After indicated treatment, the proliferation ability of BMSCs was assessed and recorded under an inverted fluorescence microscope (CKX53, OLYMPUS, Japan) according to the instructions on the EDU staining kit (C0075L, Beyotime).

### Flow analysis of apoptosis

After indicated treatment, the cells were then cultured in a 5% CO₂ atmosphere at 37°C for 48 hours. Subsequently, the treated cells were detached, gently rinsed with PBS, and incubated with 1 ml of 0.25% pancreatic enzyme. After incubation, the cellular morphology was observed under a microscope. Upon cytoplasmic retraction and loss of cell-cell connections, indicating detachment, the pancreatic enzyme was neutralized. Subsequently, 4 ml of complete media was added, and a single-cell suspension was prepared. The cell density was adjusted to 1 × 10^5^/ml. The suspension was transferred to flow tubes, and the cells were washed twice with PBS before being centrifuged at 1000 rpm for 5 minutes. Following the removal of the supernatant, the cells were resuspended in 500μl of 1 × Binding Buffer. Subsequently, 5μl of Annexin V-FITC and 10μl of PI (C1062L, Beyotime) were added to each sample as a single dye tube. The negative control group was left untreated after resuspension. After a 15-minute incubation in the dark, data acquisition was performed by a flow cytometer (CytoFLEX S, Beckman Coulter, USA).

### Lysosomal staining and imaging

After indicated treatment, the cells were then cultured in a 5% CO₂ atmosphere at 37°C for 48 hours. Subsequently, the cell culture medium was removed, and the cells were incubated with pre-prepared Lysotracker Red dyeing solution (C1046, Beyotime) at a concentration of 100nM at 37°C for 60 minutes. After rinsing with PBS, the cells were stained with 5 µg/ml of DAPI (C1002, Beyotime) for 5–10 minutes. Following another rinse with PBS, fresh cell culture medium was added. The cells were kept away from light and examined under a laser-scanning ultra-high-resolution microscope (FV3000, OLYMPUS).

### Immunofluorescence

After indicated treatment, BMSCs were washed three times with PBS for 5 minutes each. The cells were fixed with 4% paraformaldehyde for 10 minutes, followed by three washes with PBS. Subsequently, the cell membrane was permeabilized with 0.2% Triton X-100 at room temperature for 5 minutes, followed by three additional washes with PBS. The cells were then blocked with 5% BSA for 30 minutes. Following blocking, CTSD antibody (1:50, ab302649, abcam, UK) and Lamp1 antibody (1:100, ab62562, abcam), diluted with 1% BSA, were added and incubated at 37°C for 2 hours. After rinsing with PBS, Alexa Fluor®488 labeled Goat anti-mouse IgG H&L (1:200, ab150113, abcam) and Alexa Fluor® 555 labeled Goat anti-Rabbit IgG H&L (1:200, ab150078, abcam) were added and incubated at 37°C for 1 hour. After another rinse with PBS, 5 µg/ml of DAPI staining was performed for 5–10 minutes. Subsequently, the cells were rinsed with PBS, and an anti-fluorescence quencher solution was added. The cells were preserved away from light and observed under a laser-scanning ultra-high-resolution microscope (FV3000, OLYMPUS).

### Acridine orange (AO) staining

After indicated treatment, the cell culture medium was aspirated, and a pre-prepared AO dyeing solution (R20290, Yuanye Bio-Technology, Shanghai, China) was added and pre-incubated at 37°C. The final concentration of AO dye was adjusted to 15 µg/ml, gently mixed, and incubated at room temperature for 20 minutes in the absence of light. After rinsing with PBS, fresh cell culture medium was added, and the cells were examined under a laser scanning ultra-high-resolution microscope (FV3000, OLYMPUS).

### Western blot

After indicated treatment, each cell group was lysed in the corresponding RIPA lysate (P0013, Beyotime), and the lysates were incubated at 4˚C for 30 minutes. The supernatants were collected by centrifugation at 10,000 rpm for 10 minutes, and the total protein content was determined using a BCA kit (P0012, Beyotime). Protein samples were denatured, loaded onto gels, electrophoresed for 1−2 hours, and transferred onto a membrane for 30−50 minutes. The primary antibodies were incubated overnight at 4˚C, followed by incubation with secondary antibodies at room temperature for 1−2 hours. The membranes were visualized with ECL solution by using the fully-automatic chemiluminescence imager (5200, Tannon, China). The following primary antibodies were used: Anti-GAPDH (1:1000, bsm-33033M, Bioss, China), Anti-β-Tubulin (1:1000, ab18207, abcam), Anti-CD9 (1:1000, ab263019, abcam), Anti-CD63 (1:1000, ab134045, abcam), Anti-CTSD (1:1000, ab302649, abcam), Anti-BID (1:1000, ab272880, abcam), Anti-Caspase-3 (1:1000, ab184787, abcam), and Anti-Caspase-1 (1:1000, ab286125, abcam). Horseradish peroxidase (HRP)-conjugated secondary antibodies used were: Goat Anti-Rabbit IgG (1:10,000, S0001, Affinity, China) and Goat Anti-Mouse IgG (1:10,000, S0002, Affinity, China).

### Statistical analysis

Data analysis in this study was conducted using SPSS 20.0 software, and quantitative data were expressed as mean ± standard deviation. One-Way ANOVA was employed for comparing data among different study groups. P values<0.05 were considered significant.

## Results

### hUMSCs-exo@Cyasterone attenuates the proliferation inhibition and apoptosis of BMSCs induced by DEX

The identification of hUCMSC-exo and quantification of hUCMSC-exo@Cyasterone concentration can be found in S1 Fig. MTT assay was employed to assess the proliferation of BMSCs. Under normal physiological conditions, the hUMSCs-exo@Cyasterone groups exhibited minimal toxicity towards BMSCs, with the cells displaying normal growth patterns. However, in the Dex-induced model, the proliferation ability of BMSCs was significantly diminished. Nonetheless, treatment with hUMSCs-exo@Cyasterone partially restored the proliferation ability of BMSCs.

Furthermore, EDU staining demonstrated that hUMSCs-exo@Cyasterone enhanced the proliferation of the BMSC model. Dex-induced BMSCs exhibited a marked decrease in proliferation. Conversely, treatment with hUMSCs-exo@Cyasterone groups led to an enhancement in BMSC proliferation, with hUMSCs-exo@Cyasterone exerting the most pronounced effect.

Flow cytometry (FCM) analysis revealed that hUMSCs-exo@Cyasterone attenuated the apoptosis of Dex-induced BMSCs. Compared to the control group, Dex-induced BMSCs displayed a significant increase in apoptosis (****p < 0.0001). However, treatment with hUMSCs-exo@Cyasterone groups mitigated the apoptotic damage to BMSCs to a certain extent, with hUMSCs-exo@Cyasterone exhibiting the most substantial improvement.

**Fig 1:**
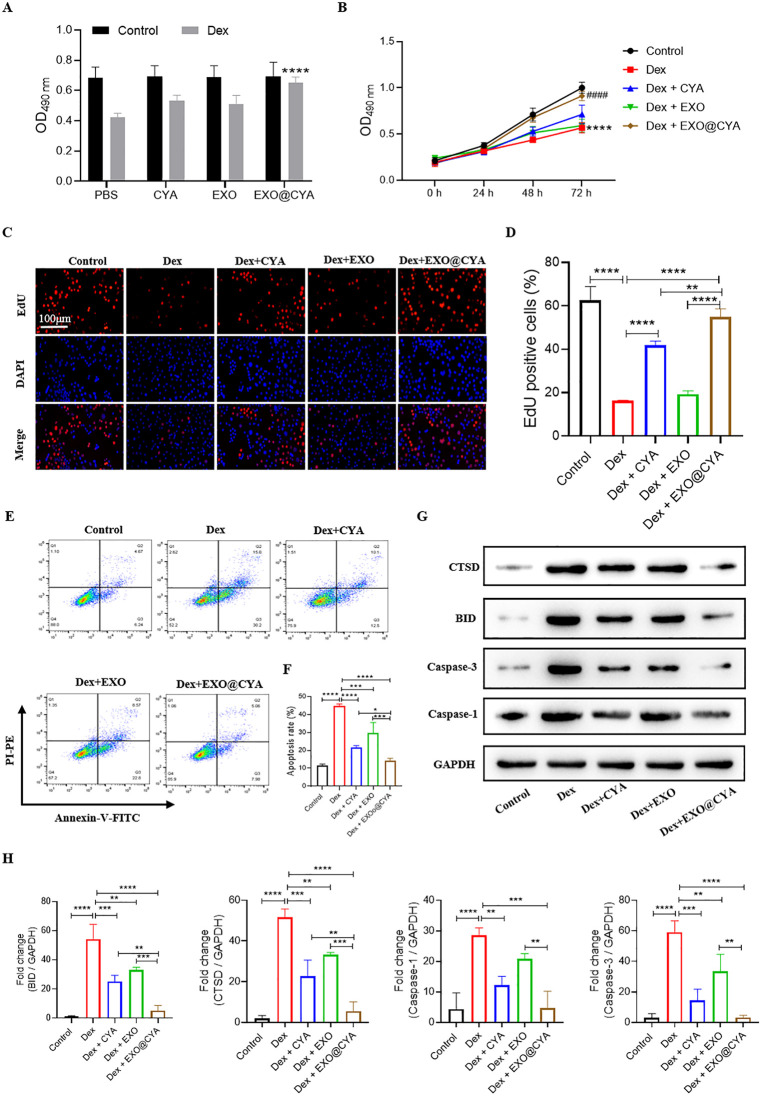
Effects of hUMSCs-exo@Cyasterone on the proliferation and apoptosis of Dex-induced BMSCs. 1A: MTT analysis indicated that hUMSCs-exo@Cyasterone had minimal impact on BMSCs toxicity and could partially reverse their proliferation ability. 1B: hUMSCs-exo@Cyasterone groups demonstrated an enhancement in the proliferation ability of Dex-induced BMSCs. 1C: EDU staining (400×) was employed to assess the effect of hUMSCs-exo@Cyasterone on BMSC model proliferation. 1D: Bar chart of statistical results showed hUMSCs-exo@Cyasterone groups exhibited an improvement in BMSCs proliferation to a certain extent. 1E: Flow cytometry analysis revealed that hUMSCs-exo@Cyasterone could mitigate the apoptosis of BMSC model. 1F: Bar chart of statistical results showed hUMSCs-exo@Cyasterone groups demonstrated a reduction in the apoptosis level of BMSCs model to a certain extent. 1G: Western blot analysis depicted that hUMSCs-exo@Cyasterone groups reduced the expression levels of apoptotic proteins. 1H: The statistical results of the fold changes of CTSD, BID, Caspase-1 and Caspase-9. Here, CYA represents Cyasterone, EXO is short for hUMSCs-exo, and EXO@CYA signifies hUMSCs-exo@Cyasterone. Statistical significance between different groups is indicated as *P < 0.05, **P < 0.01, ***P < 0.001, ****P < 0.001, *P < 0.001, n = 3.

Western blot analysis indicated that hUMSCs-exo@Cyasterone reduced the expression levels of apoptosis-related proteins in the BMSC model. In comparison to the control group, the pro-apoptotic proteins CTSD, BID, Caspase-3, and Caspase-1 were significantly upregulated in the BMSC model. However, treatment with hUMSCs-exo@Cyasterone resulted in a reduction of these proteins, indicating its potential anti-apoptotic effects. All the results in this part were showed in Fig 1.

### hUMSCs-exo@Cyasterone protects the lysosomal membrane permeability of Dex-induced BMSCs

**Fig 2:**
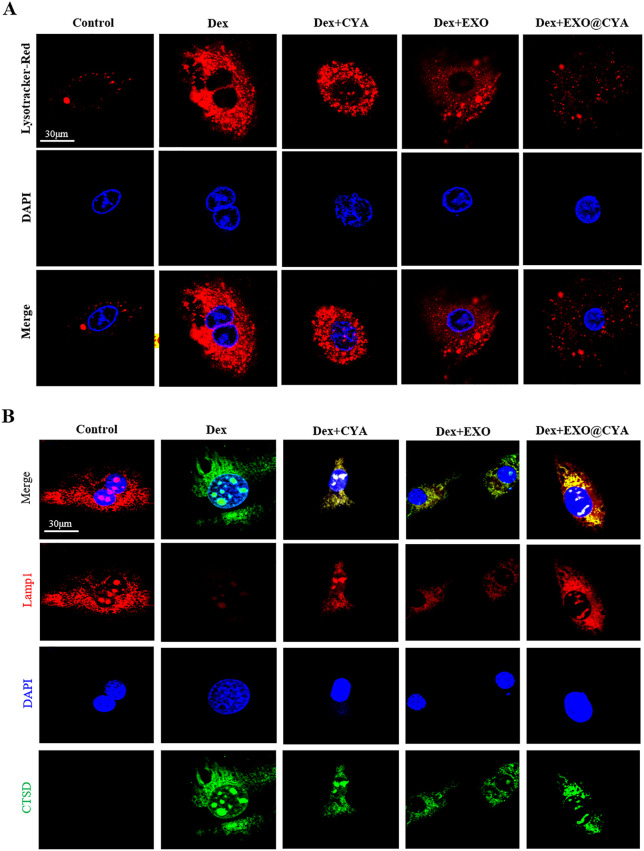
The effect of hUMSCs-exo@Cyasterone on lysosomal membrane permeability of Dex-induced BMSCs. 2A: Lysotracker Red staining was employed to assess the effect of hUMSCs-exo@Cyasterone on the lysosomal membrane permeability of BMSC models. It was observed that hUMSCs-exo@Cyasterone could reduce the permeability of lysosomal membrane to some extent (600×). 2B: AO staining was utilized to evaluate lysosomal membrane permeability in BMSCs. Confocal results demonstrated that hUMSCs-exo@Cyasterone could mitigate the change in lysosome permeability to a certain extent (600 × , with decreased green fluorescence and increased red fluorescence). Transfection with CTSD-WT weakened the protective effect of hUMSCs-exo@Cyasterone on lysosomal membrane permeability, whereas CTSD-N258A enhanced this protective effect.

Lysotracker Red staining showed that hUMSCs-exo@Cyasterone could reduce the permeability of lysosomal membrane in Dex-induced BMSCs. The AO staining and Confocal results demonstrated that hUMSCs-exo@Cyasterone could mitigate the change of CTSD in lysosome permeability to a certain extent in Dex-induced BMSCs (Fig 2).

### hUMSCs-exo@Cyasterone inhibits the expression level of the Lysosomal localization of CTSD in Dex-induced BMSCs

**Fig 3:**
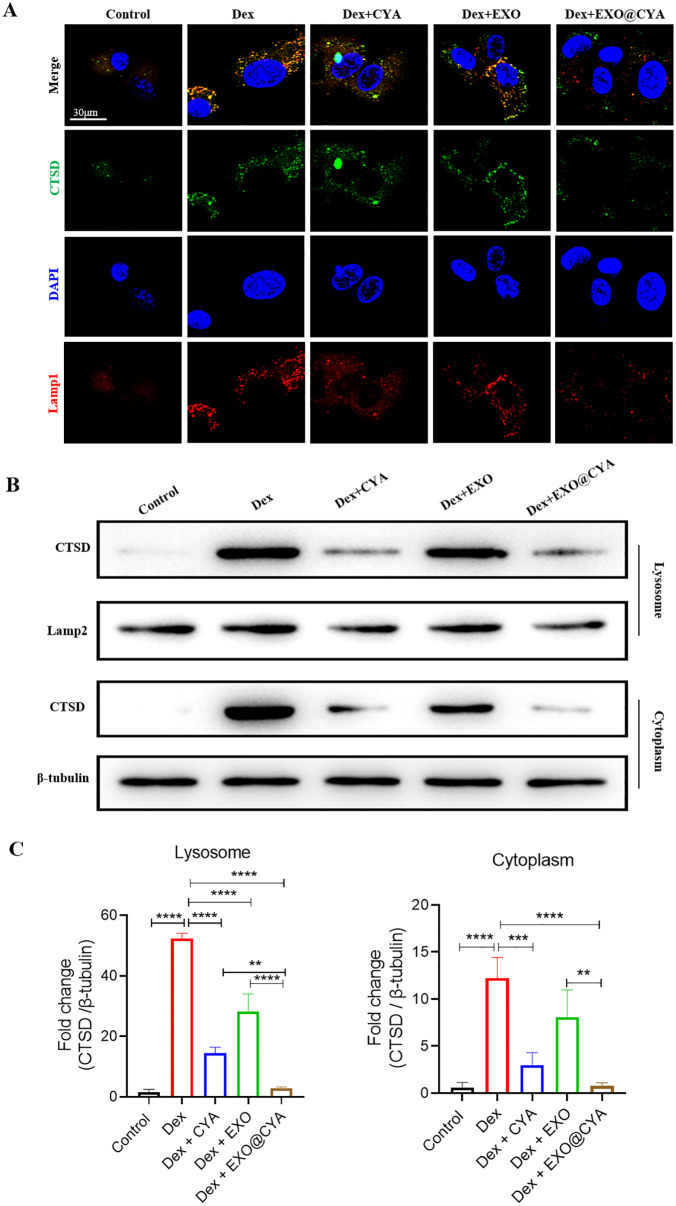
Effects of hUMSCs-exo@Cyasterone on the lysosomal localization of CTSD protein in Dex-induced BMSCs. 3A: Both CTSD and Lamp1 were significantly up-regulated in the BMSC model, with co-localization observed between CTSD and Lamp1 (600×). All groups treated with hUMSCs-exo@Cyasterone exhibited a decrease in the expression levels of CTSD and Lamp1, and inhibited the expression of CTSD in lysosomes, with hUMSCs-exo@Cyasterone showing the strongest inhibitory effect. 3B: CTSD demonstrated consistent changes in both lysosome and cytoplasm. 3C: The statistical results of the fold changes of CTSD in lysosome and cytoplasm. Here, CYA represents Cyasterone, EXO is short for hUMSCs-exo, and EXO@CYA signifies hUMSCs-exo@Cyasterone. Statistical significance between different groups is indicated as *P < 0.05, **P < 0.01, ***P < 0.001, ****P < 0.001, *P < 0.001, n = 3.

Immunofluorescence analysis revealed that hUMSCs-exo@Cyasterone decreased the expression levels of CTSD and Lamp1 in the BMSC model. Compared to the Control group, both CTSD and Lamp1 (lysosome-associated membrane protein 1) were significantly up-regulated in the BMSC model, and CTSD and Lamp1 exhibited co-localization. Treatment with hUMSCs-exo@Cyasterone resulted in decreased expression levels of CTSD and Lamp1 across all groups, inhibiting CTSD expression in lysosomes, with hUMSCs-exo@Cyasterone exhibiting the most pronounced inhibitory effect. Western blot analysis demonstrated consistent changes in CTSD and Lamp2 expression levels in the cytosol and lysosomes. All the results in this part were showed in Fig 3.

### Dex promotes the N-glycosylation of CTSD in BMSCs

**Fig 4:**
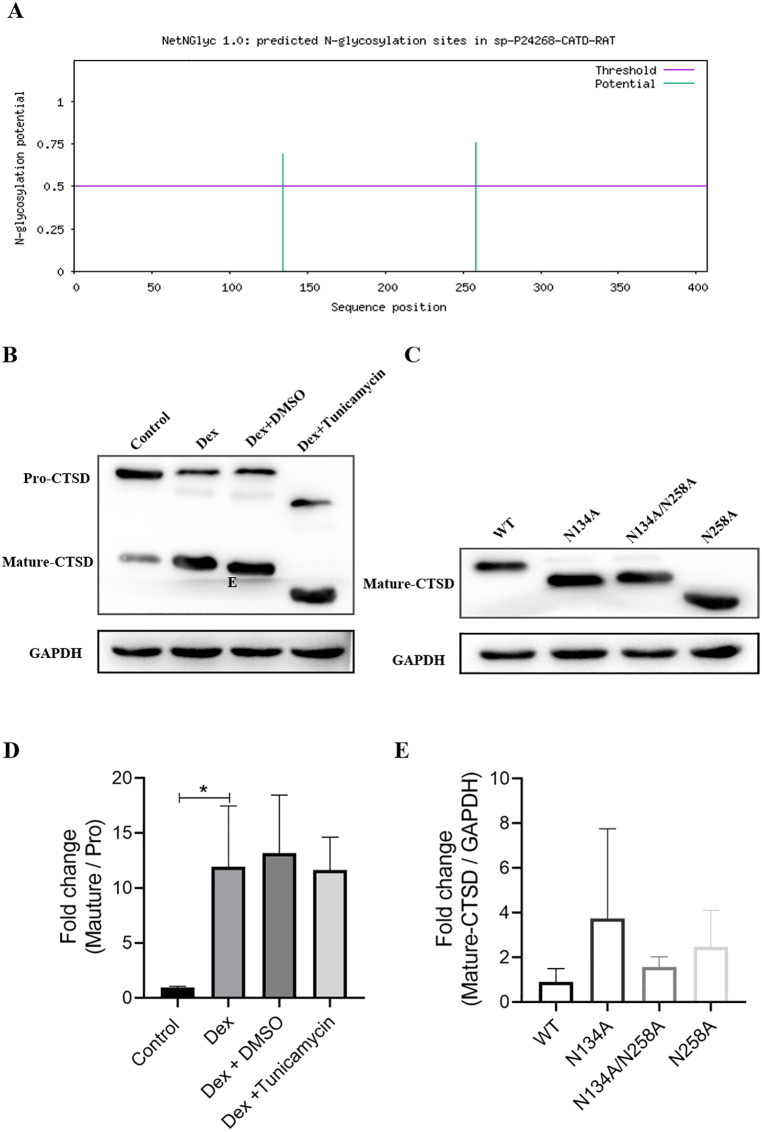
Dex promotes N-glycosylation of CTSD protein in BMSCs. 4A: Bioinformatics predicted two N-glycosylation modification sites in rat CTSD, resulting in three N-glycosylation modification mutants, namely N258A and N134A. 4B: Western blot analysis demonstrated that Tunicamycin reduced the molecular weight of CTSD to some extent. 4C: Western blot analysis showed a decrease in the molecular weight of mature CTSD after transfection with N-glycosylated mutants N134A, N258A, or N134/N258A of CTSD. Among them, the N258A mutant exerted the greatest influence on the molecular weight of mature CTSD. 4D: The statistical results of the fold change of mature CTSD compared with pro-CTSD. 4E: The statistical results of the fold change of mature CTSD in N134A, N258A and N134/N258A.

Bioinformatics analysis predicted two N-glycosylation sites in rat CTSD. Based on this prediction, three mutants were generated: N134A, N258A, and N134A/N258A. After transfection with the N-glycosylation site mutants of CTSD (N134A, N258A, and N134A/N258A), the molecular weight of mature CTSD (m-CTSD) was found to be decreased compared to the WT group in western blot analysis. This indicates that dexamethasone promotes the N-glycosylation of CTSD in BMSCs. Notably, among these mutants, N258A had the most significant impact on the m-CTSD’s molecular weight. All the results in this part were showed in Fig 4.

### CTSD-N258A enhances the effect of hUMSCs-exo@Cyasterone on the proliferation and apoptosis of Dex-induced BMSCs

**Fig 5:**
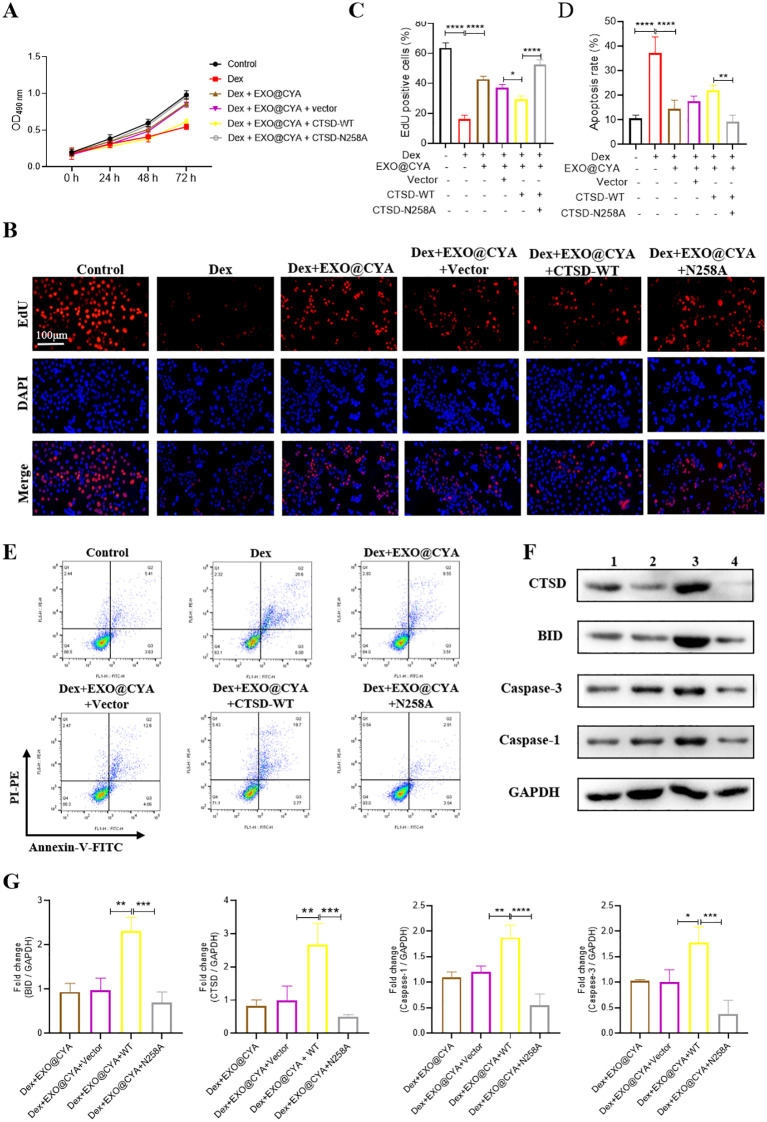
CTSD-N258A enhances the effect of hUMSCs-exo@Cyasterone on the proliferation and apoptosis of Dex-induced BMSCs. 5A: MTT analysis indicated that CTSD-N258A is involved in hUMSCs-exo@Cyasterone-mediated protection. 5B: Edu staining (400×) was used to analyze BMSC proliferation, revealing that hUMSCs-exo@Cyasterone partially mitigated the proliferation of Dex-induced BMSCs. 5C: Bar chart of statistical results showed CTSD-N258A enhanced the proliferative protection of hUMSCs-exo@Cyasterone on BMSCs. 5D: The bar chart of the statistical results of the apoptosis rate of CTSD-N258A in BMSC model. 5E: Flow cytometry analysis demonstrated that N-glycosylated CTSD-N258A inhibited the apoptosis level of the BMSC model to a certain extent. 5F: Western blot analysis revealed that CTSD-N258A inhibited the expression of apoptotic proteins to a certain extent. 5G: The statistical results of the fold changes of the CTSD, BID, Caspase-1 and Caspase-9 in CTSD-N258A treated BMSC model. Here, CYA represents Cyasterone, EXO is short for hUMSCs-exo, and EXO@CYA signifies hUMSCs-exo@Cyasterone. In addition, 1: Dex + EXO@CYA, 2: Dex + EXO@CYA+Vector, 3: Dex + EXO@CYA + WT, 4: Dex + EXO@CYA + N258A. Statistical significance between different groups is indicated as *P < 0.05, **P < 0.01, ***P < 0.001, ****P < 0.001, *P < 0.001, n = 3.

MTT assay was employed to assess whether N-glycosylated mutant CTSD-N258A influenced the protective effect of hUMSCs-exo@Cyasterone. The results demonstrated that CTSD-N258A indeed affected the protective effect mediated by hUMSCs-exo@Cyasterone. In comparison to CTSD-WT, CTSD-N258A significantly promoted the proliferation of BMSCs. Additionally, Edu staining revealed that, relative to the Control group, the proliferation level of the BMSC model significantly decreased. However, hUMSCs-exo@Cyasterone mitigated the proliferation impairment of Dex-induced BMSCs to a certain extent. Furthermore, CTSD-WT attenuated the proliferative effect of hUMSCs-exo@Cyasterone on BMSCs, whereas CTSD-N258A enhanced this protective effect. Flow cytometry analysis was conducted to evaluate whether N-glycosylation-modified mutants of CTSD affected the protective effect mediated by hUMSCs-exo@Cyasterone. In comparison to the CTSD-WT group, CTSD-N258A was found to partially inhibit the apoptosis level of the BMSC model. Western blot analysis was employed to assess the effects of CTSD-N258A on apoptosis-related proteins in BMSC models. Relative to the vector group, CTSD-WT transfection further upregulated the expression of apoptotic proteins, whereas CTSD-N258A inhibited the expression of apoptotic proteins to some extent. All the results in this part were showed in Fig 5.

### CTSD-N258A enhances the protective effect of hUMSCs-exo@Cyasterone on lysosomal membrane permeability in Dex-induced BMSCs

**Fig 6:**
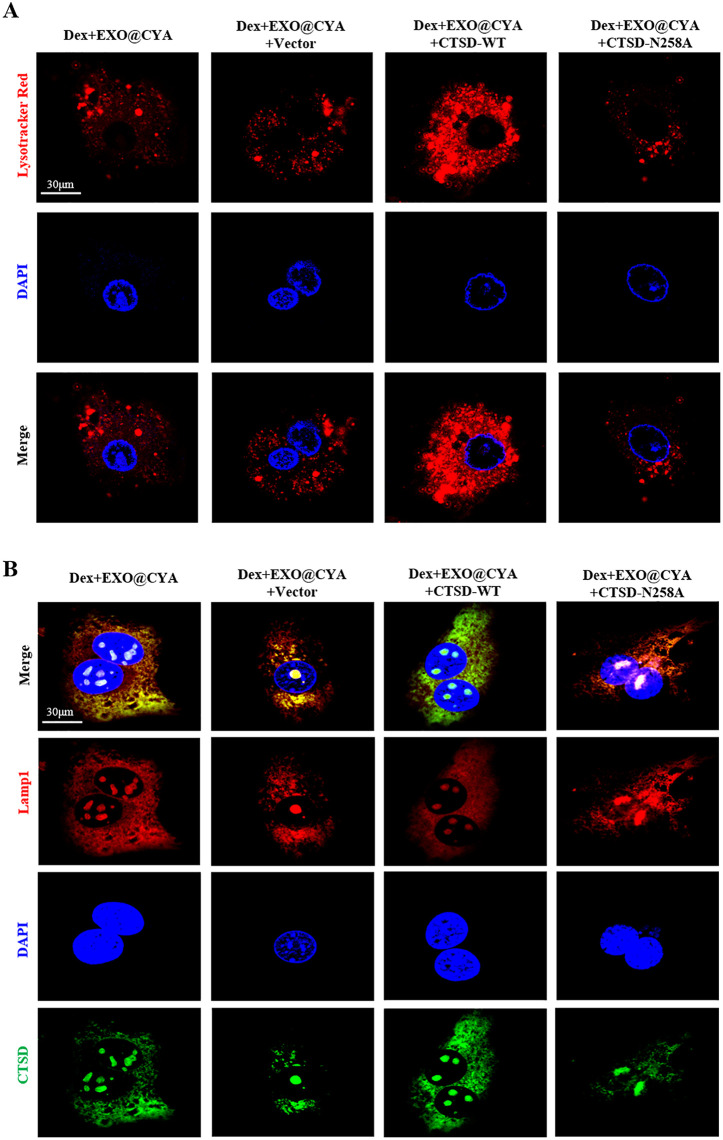
CTSD-N258A enhances the protective effect of hUMSCs-exo@Cyasterone on lysosomal membrane permeability in Dex-induced BMSCs. 6A: Lysotracker Red staining was employed to analyze lysosomal membrane permeability in BMSCs. The results demonstrated that hUMSCs-exo@Cyasterone could reduce lysosomal membrane permeability to some extent. Furthermore, CTSD-N258A increased the protective effect of hUMSCs-exo@Cyasterone, as indicated by weakened red fluorescence (600×). 6B: AO staining was performed to assess lysosomal permeability in the BMSC model treated with hUMSCs-exo@Cyasterone. The results revealed decreased lysosomal membrane stability induced by Dex, accompanied by enhanced cytoplasmic green fluorescence (600×). Moreover, hUMSCs-exo@Cyasterone groups exhibited inhibition of lysosomal permeability to a certain extent.

Lysotracker red staining was utilized to analyze the lysosomal membrane permeability in BMSCs. Compared to the Control group, the lysosomal fluorescence intensity of BMSCs induced by Dex increased, indicating enhanced lysosomal membrane permeability. However, hUMSCs-exo@Cyasterone partially reduced the permeability of the lysosomal membrane. Moreover, CTSD-WT attenuated the protective effect of hUMSCs-exo@Cyasterone on lysosomal membrane permeability (indicated by enhanced red fluorescence), while CTSD-N258A augmented this protective effect (indicated by reduced red fluorescence). AO staining was employed to analyze the effect of hUMSCs-exo@Cyasterone on lysosomal permeability in the BMSC model. As depicted, the lysosomal membrane stability of BMSCs induced by Dex was compromised, as evidenced by increased cytoplasmic green fluorescence. Additionally, hUMSCs-exo@Cyasterone groups exhibited inhibition of lysosomal permeability to some extent. Among them, hUMSCs-exo@Cyasterone exerted the most potent inhibitory effect on lysosome permeability. All the results in this part were showed in Fig 6.

### CTSD-N258A promotes the inhibition effect of hUMSCs-exo@Cyasterone on lysosomal localization of CTSD in Dex-induced BMSCs

**Fig 7:**
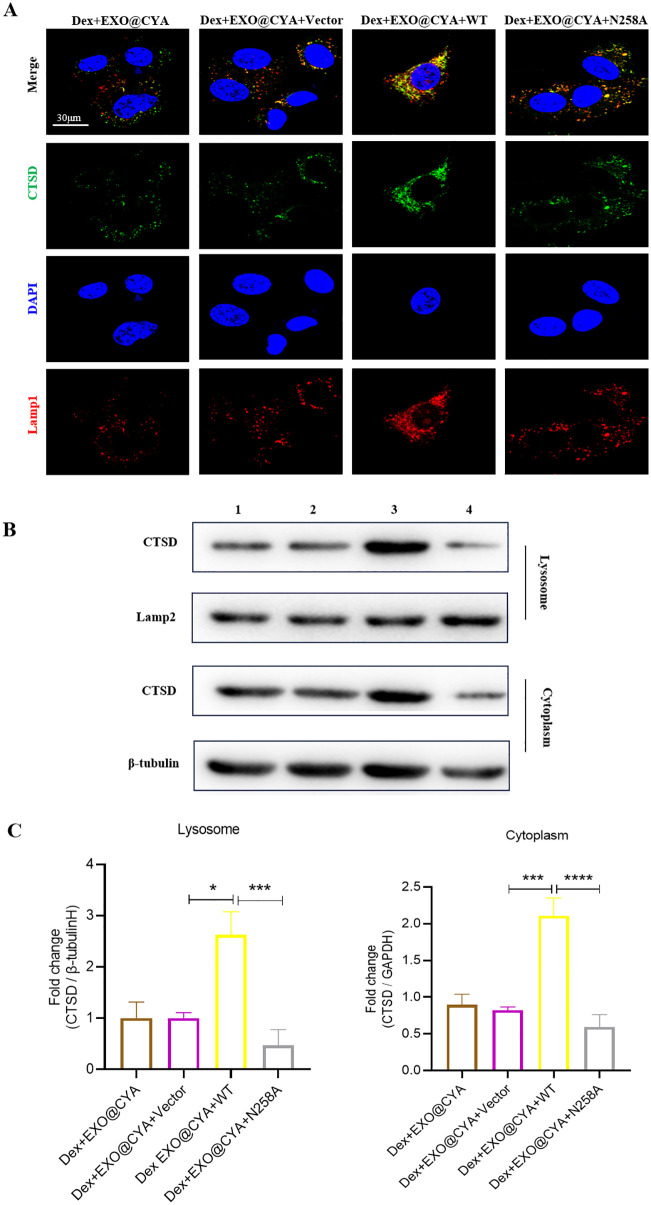
CTSD-N258A enhances the inhibitory effect of hUMSCs-exo@Cyasterone on the lysosomal localization of CTSD in Dex-induced BMSCs. 7A: The lysosomal localization of CTSD in the BMSC model was observed through immunofluorescence. Compared with the vector group, CTSD-WT transfection further promoted the expression of CTSD and Lamp1, with a certain degree of co-localization observed between CTSD and Lamp1. In contrast, transfection of CTSD-N258A resulted in decreased expression of CTSD and Lamp1 (600×). 7B: Western blot analysis revealed that transfection with CTSD-N258A decreased the expression of CTSD. 7C: The statistical results of the fold changes of the CTSD protein level in CTSD-N258A treated BMSC model in lysosome and cytoplasm. Here, CYA represents Cyasterone, EXO is short for hUMSCs-exo, and EXO@CYA signifies hUMSCs-exo@Cyasterone. In addition, 1: Dex + EXO@CYA, 2: Dex + EXO@CYA+Vector, 3: Dex + EXO@CYA + WT, 4: Dex + EXO@CYA + N258A. Significant differences between different groups are indicated as *P < 0.05, **P < 0.01, ***P < 0.001, ****P < 0.001, *P < 0.001, n = 3.

The lysosomal localization of CTSD in the BMSC model was observed via immunofluorescence analysis. In comparison to the vector group, CTSD-WT transfection markedly enhanced the expression of CTSD and Lamp1, and a certain degree of co-localization between CTSD and Lamp1 was observed. Conversely, transfection with CTSD-N258A led to a decreased expression of CTSD and Lamp1. Western blot analysis was employed to investigate the impact of CTSD-N258A and hUMSCs-exo@Cyasterone on CTSD expression in lysosomes in the BMSC model. Relative to the vector group, CTSD-WT transfection significantly upregulated the expression of CTSD in both the cytoplasm and lysosomes, while transfection with CTSD-N258A resulted in decreased expression of CTSD. All the results in this part were showed in Fig 7.

## Discussion

Exosome-based drug delivery represents an emerging strategy for treating steroid-induced osteonecrosis of the SIONFH. It utilizes exosomes as natural nanocarriers to efficiently load and target therapeutic molecules, such as anti-apoptotic drugs, pro-angiogenic factors, or specific miRNAs, to the ischemic necrotic femoral head region [23,24]. Although SIONFH pathogenesis remains incompletely understood, pivotal contributing factors include abnormal differentiation of BMSCs and osteoblasts, apoptosis of BMSCs, osteoblasts and osteoclasts, the lipid metabolism disorders, and coagulation pathway disorders. Apoptosis is considered crucial in SIONFH occurrence and progression of SIONFH, suggesting that inhibiting apoptosis may help prevent SIONFH, even in its Dex-induced BMSCs model. This study demonstrates that hUMSCs-exo@Cyasterone ameliorates Dex-induced apoptosis in BMSCs, providing new insights for its potential clinical application and strong evidence for exploring exosome-loaded natural compounds in SIONFH treatment.

Exosomes leverage their excellent biocompatibility, low immunogenicity, and inherent homing ability to overcome blood supply barriers in lesion sites, enabling precise drug delivery to bone cells [25]. Simultaneously, drug-loaded exosomes—particularly stem cell-derived variants—exert synergistic therapeutic effects including anti-inflammatory, anti-apoptotic, pro-angiogenic, and bone-repair properties, demonstrating significant necrotic bone tissue repair potential in preclinical studies [26].

Exosome-based drug delivery technology offers compelling pharmaceutical applications, such as enhanced circulatory bioavailability, targeted delivery capabilities, and reduced immune reactions [27],with additional benefits of minimal hepatotoxicity and broad biocompatibility enabling utility across medical domains [28]. Notably, hUCMSC-exo exhibit protective effects against SIONFH by inhibiting osteocytes and BMSCs apoptosis [29]. Our study further confirms these advantages by showing that hUCMSC-exo@Cyasterone is more effective than Cyasterone alone at reducing Dex-induced BMSC apoptosis and inhibiting lysosomal membrane permeability, highlighting the value of exosome-based delivery.

Glycosylation, a key modification regulating CTSD functionality and stability, directly affecting its activity and localization within lysosomes [30]. However, the role of CTSD in apoptosis is complex, as it might modulate apoptosis and proliferation under diverse physiological and pathological conditions.

hUMSCs-exo may regulate apoptosis by influencing the glycosylation of CTSD [31]. Previous studies indicate that loading natural compounds, such as resveratrol and curcumin into hUMSCs-exo enables effective delivery to target cells, thereby directly or indirectly modulating CTSD activity and expression [32,33]. This strategy not only enables precise delivery of natural compounds but may also balance cell survival and apoptosis by modulating CTSD glycosylation, offering novel insights into apoptosis regulation and therapeutic strategies for related diseases [34]. Potential mechanisms for this modulation impacting apoptosis: hUMSCs-exo loaded with natural compounds downregulate CTSD glycosylation, inhibiting its overactivation and thus reducing apoptotic signaling, which may protect against inflammation- or oxidative stress-induced cell damage; conversely, in pathological or tumor cells, specific natural compounds loaded into hUMSCs-exo may enhance CTSD glycosylation, increasing its lysosomal stability or activity, thereby amplifying apoptotic signaling and promoting damaged cell clearance [35].

CTSD N-glycosylation exhibits context-dependent roles influenced by N-glycan structure, species, and tissue specificity. Previous research established that N-glycosylation is essential for CTSD’s correct lysosomal localization, a finding corroborated by our experiments: transfection with the N-glycosylation mutant N258A reduced mature CTSD expression levels [36,37]. Notably, CTSD-N258A significantly promoted BMSC proliferation and partially inhibited BMSC apoptosis according to flow cytometry. Existing literature indicates that glycosylation at asparagine 233 (N233) regulates pro-CTSD secretion, while ecdysone promotes CTSD expression, induces maturation via autophagy, and facilitates Caspase-3 activation to drive midgut cell apoptosis [16]. Both human and rat CTSD proteins contain predicted N-glycosylation sites that govern zymogen secretion, maturation, and apoptosis induction. Collectively, these data confirm that CTSD N-glycosylation modulates BMSC apoptosis, though its specific regulatory mechanisms in steroid-induced BMSC apoptosis remain unresolved. Our data shows that hUCMSC-exo@Cyasterone downregulates CTSD-related protein expression. Moreover, the CTSD-N258A variant was found to inhibit BMSC apoptosis (a decreased expression of CTSD and Lamp1), augment the cytoprotective effect of hUCMSC-exo@Cyasterone, and enhance CTSD’s lysosomal localization while reducing lysosomal membrane permeability.

**Fig 8:**
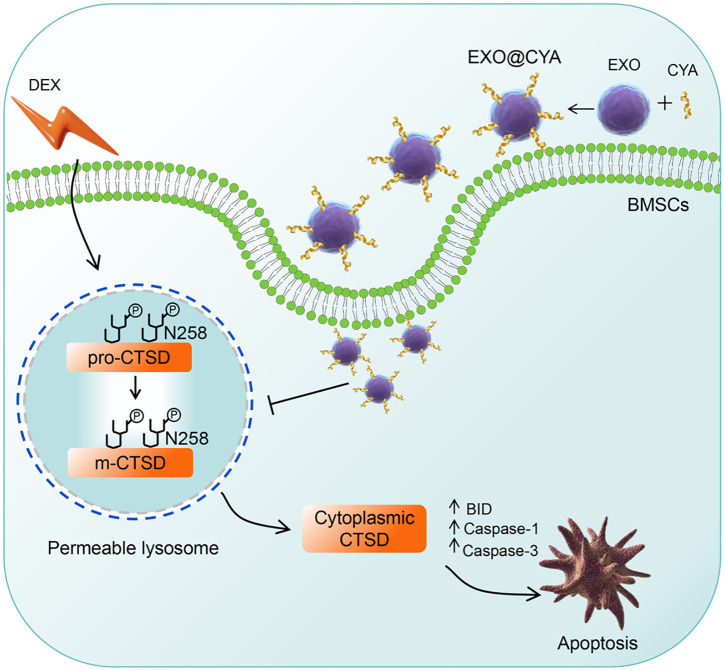
The graphic abstract of the protective effect of hUMSCs-exo@Cyasterone in Dex-induced BMSCs. Dex promoted the lysosomal membrane permeability by up-regulating the pro-CTSD and m-CTSD, thus to increase the protein expression level of cytoplasmic CTSD and finally resulted in an improved apoptosis-related protein expression levels of BID, Caspase-1 and Caspase-9. However, hUMSCs-exo@Cyasterone ameliorated this pathological state. This graphic abstract showed hUMSCs-exo@Cyasterone alleviated the cellular model, the Dex-induced BMSCs, through the N-glycosylation modulation of CTSD (specifically CTSD-N258A). Here, m-CTSD is short for mature-CTSD.

Taken together, our study demonstrated that the exosome-loaded compound, hUMSCs-exo@Cyasterone, alleviated the cellular model of SIONFH disease through the CTSD-N258A. The graphic abstract of the protective effect of hUMSCs-exo@Cyasterone in Dex-induced BMSCs is shown in Fig 8. The current study has potential limitations. It was conducted entirely in vitro, and there is a lack of direct biochemical validation of CTSD glycosylation. Furthermore, these limitations need to be addressed through additional further in vitro and in vivo experiments.

## Conclusions

In conclusion, this study has shown that hUMSCs-exo@Cyasterone could regulate the N-glycosylation modification of CTSD (specifically CTSD-N258A) in Dex-induced BMSCs (a cellular model of steroid-induced femoral head necrosis). The findings of this research might be helpful for the potential clinical utilization of hUMSCs-exo@Cyasterone and contribute novel evidence to the exploration of exosome-loaded natural compounds in the treatment of SIONFH disease.

## Supporting information

S1 Fig Identification of hUCMSC-exo and quantification of hUCMSC-exo@Cyasterone concentration. (A) Morphology of passage 4 (P4) hUCMSCs observed under an inverted microscope. Scale bar = 500μm. (B) Western blot analysis of exosomal protein markers (CD9 and CD63) in hUCMSC-exo. (C) Representative transmission electron microscopy (TEM) images of hUCMSC-exo. Scale bar = 300nm. (D) Determination of hUMSCs-exo particle size using Malvern Particle Size Analyzer.
(TIF)

S1 File Raw_images. (RAR)
