# Supplementary figures and images for: hUMSCs-exo@Cyasterone protects the cell model of steroid-induced femur head necrosis by regulating N-glycosylation modification of CTSD-N258A
Source: PLoS One. 2026 Apr 3;21(4):e0337562. doi: 10.1371/journal.pone.0337562 (PMC13048378; doi:10.1371/journal.pone.0337562)

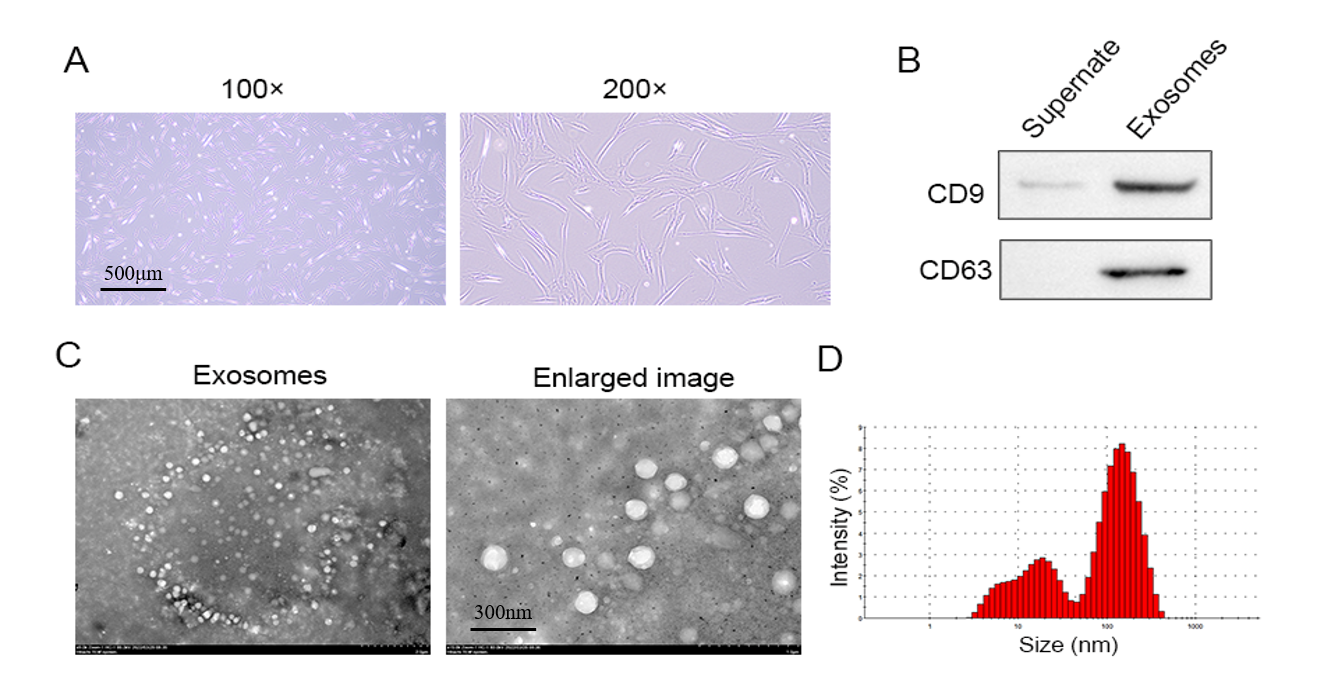

Supplement: S1 Fig — (A) Morphology of passage 4 (P4) hUCMSCs observed under an inverted microscope. Scale bar = 500μm. (B) Western blot analysis of exosomal protein markers (CD9 and CD63) in hUCMSC-exo. (C) Representative transmission electron microscopy (TEM) images of hUCMSC-exo. Scale bar = 300nm. (D) Determination of hUMSCs-exo particle size using Malvern Particle Size Analyzer. (TIF) [file pone.0337562.s001.tif]
